# Mobile photon counting detector CT with multi material decomposition methods for neuroimaging of patients in intensive care unit

**DOI:** 10.1038/s41598-024-81735-x

**Published:** 2024-12-30

**Authors:** Su-Jin Park, Kwanhee Han, Junyoung Park, Jonghwan Min, Dufan Wu, Doil Kim, Kyutae Kang, Duhgoon Lee, Rajiv Gupta, Jinwook Jung

**Affiliations:** 1 Health & Medical Equipment Business, Samsung Electronics Co., Ltd, 8, Gumi-ro, Bundang-gu, Seongnam- si, 13638 Gyeonggi-do Republic of Korea; 2 Massachusetts General Hospital, 55 Fruit St., Radiology, Boston, MA 02114 USA; 3 Samsung NeuroLogica, 14 Electronics Ave, Danvers, MA 01923 USA

**Keywords:** X-ray tomography, Imaging techniques

## Abstract

The photon-counting detector computed tomography (PCD-CT) is a promising new technology that provides more spectral information in medical imaging. PCD-CT enables bedside imaging in the neuro intensive care unit (neuro ICU) for patients with life-threatening conditions such as brain hemorrhage and ischemic stroke. The primary purpose of this study is to evaluate a multi-material decomposition algorithm available on PCD-CT, dubbed *MD Plus*, to differentiate between contrast agent and hemorrhage in hyperdense lesions. A certified multi-energy phantom was used to validate its performance with various x-ray exposure conditions and locations of contrast agent. The results from the quantitative analysis of multi-energy phantoms and the clinical cases of patients in the ICU demonstrated that MD Plus can accurately differentiate between the contrast agent and the hemorrhage. The extended MD Plus algorithm, including virtual non-contrast (VNC) and bone removal, was also validated for various clinical applications.

## Introduction

Computed tomography (CT) brain examinations are a frequently used imaging modality in patients with symptoms of intracranial hemorrhage and acute brain stroke^1–3^. The primary focus of this paper is a mobile PCD-CT scanner, designed to bring imaging close to the bedside to avoid risks associated with transporting critically ill patients to a fixed CT scanner in an imaging suite. The scanner can be easily moved around the hospital from one room to another or an operating room for intra-operative imaging. In the intensive care unit (ICU), the mobile PCD-CT allows early neuroimaging avoiding many of the risks associated with the transportation of critically ill patients with life-threatening conditions such as brain hemorrhage and ischemic stroke^4^.

The PCD-CT is an advanced CT technique using more than two separate X-ray photon energy spectra, which allows the distinction of materials with different attenuation properties at different energies. The mobile PCD-CT investigated in this paper became clinically available after FDA clearance in February 2022. A PCD-CT has higher spatial resolution and more spectral information compared to the conventional energy-integrated detector (EID)-CT^5–7^. Multi-spectral imaging provides additional capabilities for CT analysis, namely material decomposition and virtual monoenergetic images (VMI).

In addition, the capability of material decomposition to handle a more extensive list of materials extends it to more clinical applications, such as virtual non-contrast (VNC) and bone removal. The VNC provides the user with images similar to true unenhanced images. The VNC image can be obtained by decomposing the subject into iodine and non-iodine components and imaging from the non-iodine components. This technology can potentially omit plain (non-contrast) scans, thus reducing the radiation exposure^8^. Another clinical application of the material decomposition is bone removal. The bone removal application is also helpful in detecting acute intracranial hemorrhagic lesions such as subdural, epidural, or intraparenchymal hematomas and acute venous thrombosis^9,10^.

However, prior research has addressed capabilities such material decomposition and VMI^11^ assuming that the human body consists of only two or at most three materials. However, that assumption can be challenging since the human body comprises many materials, including soft tissue, blood, bone, air, water, and contrast agent.

Multi-material decomposition (MMD) methods^12^ have been proposed to extend material decomposition to more than three materials. Along these lines, we have developed a MMD algorithm (MD Plus) and optimized it for the mobile PCD-CT. The selection of material maps, such as iodine, water, and calcium, is based on clinical applications to answer questions that are not possible on a conventional CT scanner. For example, significant amount of iodine contrast agent is used in neuro-patients treated with thrombectomy. Due to damage to the blood-brain barrier in the infarcted tissue, a possibility of injury during thrombectomy, there may be staining of brain parenchyma with contrast agent. Such areas in conventional CT will appear as a hyperdense region may be indistinguishable from hemorrhagic conversion of stroke. Such a finding could result in diagnostic uncertainty, misdiagnosis, and delayed treatment such as anticoagulation. Sometimes, these patients are too sick to travel to a fixed dual or multi-spectral scanner. A more accessible CT platform to distinguish between hemorrhage and iodine contrast agent is needed. This paper presents early evidence such a distinction can be made using mobile PCD-CT platform^1,13,14^. In addition, VNC and bone removal images can be generated using our MD Plus maps.

In this study, we evaluated our MD Plus algorithm with mobile PCD-CT for neuro-patients in the ICU. We tested the characterization and differentiation of materials, such as iodine contrast and hemorrhage. Differentiation of iodine contrast and hemorrhage or acute ischemic stroke may be helpful in preexisting or incidentally detected lesions. We will also evaluate the suitability of extended MD Plus algorithm, including VNC and bone removal, for clinical applications.

## Methods

### PCD-CT systems

**Fig. 1:**
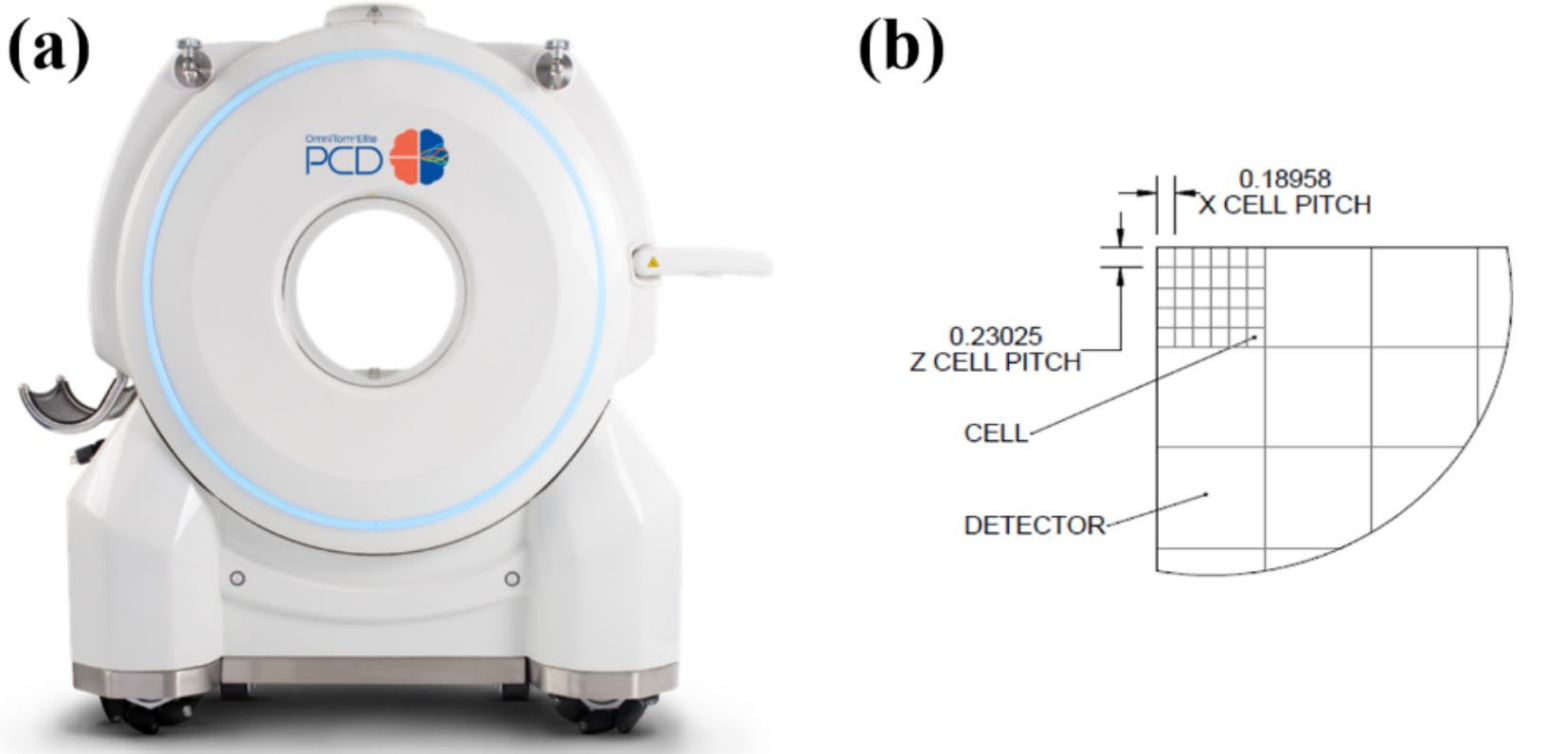
PCD is installed in (**a**) mobile CT (OmniTom Elite) and (**b**) its pixel layout.

This study used the FDA 510(k) cleared mobile PCD-CT (OmniTom Elite PCD, Samsung-Neurologica, Danvers, MA, USA). The OmniTom Elite with PCD has a cadmium telluride-based PCD (Fig. 1(a)). The PCD array comprises individual cells that are 1.4 mm thick and 0.19 × 0.23 mm^2^ (Fig. 1(b)). Special ASICs (application-specific integrated circuits) are optimized to handle the array of large data flow. The PCD array can generate spectral CT images at three energy levels: low-energy, medium-energy, and high-energy. The energy thresholds were set as 30, 40, and 50 keV. The OmniTom Elite PCD is supported for adult imaging for anatomy that can be imaged in the 40 cm aperture, primarily the head and neck.

### MMD algorithm

For the material decomposition, we used the MMD algorithm^12^ and optimized the algorithm for the mobile PCD-CT, namely MD Plus, which extends traditional material decomposition to allow for clarification and visualization of many materials. The key idea to extend the material map to more than two material maps is based on the assumption that the material mixture in the human body is composed of predefined materials. We created triplets of predefined materials for MD Plus and utilized this information to perform multi-material decomposition. The specific steps are listed below.

First, a primary material decomposition is performed with the following Eq. 

1 $$\:\mu\:\left(\underset{\_}{x},\:{E}_{j}\right)={\sum\:}_{m=1}^{M}{w}_{m}\left(\underset{\_}{x}\right){\mu\:}_{m}\left({E}_{j}\right)$$

where $\:\mu\:\left(\underset{\_}{x},\:{E}_{j}\right)$ is the spatial distribution of energy-dependent linear attenuation coefficients(LAC) of energy j, $\:{w}_{m}\left(\underset{\_}{x}\right)$ is the spatial distribution of material m with respect to a fraction-by-weight, $\:\mu\:\text{m}\left({E}_{j}\right)$ is linear attenuation coefficient of energy j for material m, $\:M$: the number of basis material. Using the least square method, we can solve for $\:{w}_{m}\left(\underset{\_}{x}\right)$ with given $\:\mu\:\left(\underset{\_}{x},\:{E}_{j}\right)$, and $\:\mu\:\text{m}\left({E}_{j}\right)$. We are using three basis materials: iodine, calcium, and water from the Gammex Multi-Energy CT phantom.

Second, we can find the $\:\mu\:\left(\underset{\_}{x},\:{\text{E}}_{\text{j}}\right)$ with solved $\:{w}_{m}\left(\underset{\_}{x}\right)$ as the basis material’s distribution and $\:\mu\:\text{m}\left({\text{E}}_{\text{j}}\right)$ as the mass attenuation coefficient of specific mono energy for the virtual monochromatic image (VMI).

Third, the MD Plus method was used to estimate the volume fraction in a mixture containing two or more materials. Two VMIs are used to perform MD Plus. The volume fractions of three types of substances can be estimated using the following.

2 $$\:\left[\begin{array}{ccc}{\mu\:}_{E1,m1}&\:{\mu\:}_{E1,m2}&\:{\mu\:}_{E1,m3}\\\:{\mu\:}_{E2,m1}&\:{\mu\:}_{E2,m2}&\:{\mu\:}_{E2,m3}\\\:1&\:1&\:1\end{array}\right]\left[\begin{array}{c}{a}_{m1}\\\:{a}_{m2}\\\:{a}_{m3}\end{array}\right]=\left[\begin{array}{c}{\mu\:}_{E1}\\\:{\mu\:}_{E2}\\\:1\end{array}\right]$$

**Fig. 2:**
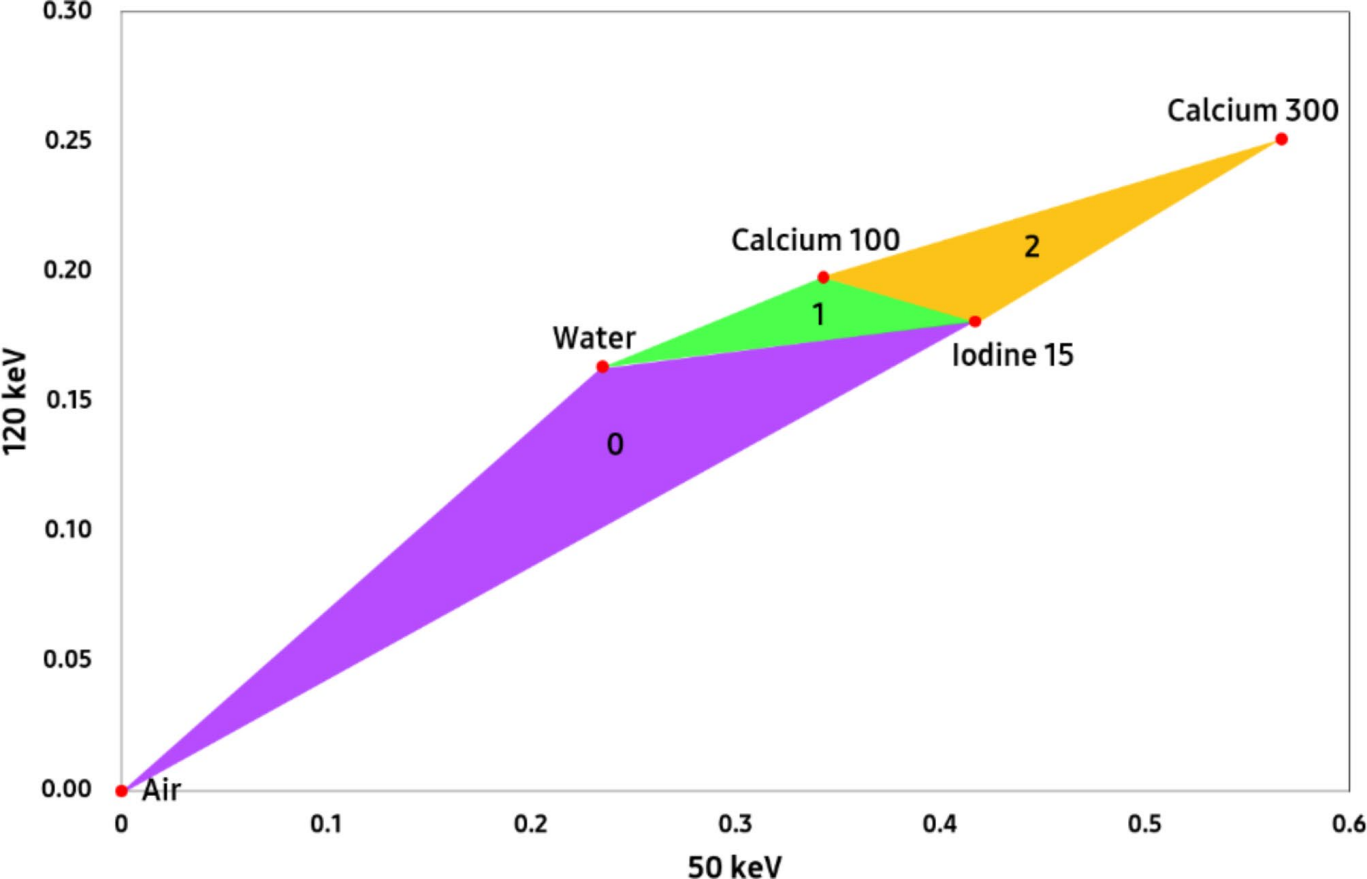
The triangle set for MD Plus was created using LAC of ‘Air,’ ‘Water,’ ‘Iodine 15 mg/cc’, ‘Calcium 100 mg/cc’, and ‘Calcium 300 mg/cc’ as the vertices, obtained experimentally from Gammex phantom or using theoretical values from NIST.

It is assumed that the materials within the human body form a mixed substance with three defined materials and that the sum of volume fractions must be 1. $\:{\mu\:}_{E1}$ and $\:{\mu\:}_{E2}$ represent the linear attenuation coefficient (LAC) of the spatial distribution in mono energy. E1 and E2 have to be fixed at specific mono-energy pairs (ex. 50 keV and 120 keV). The spatial distribution of material m with respect to fraction-by-volume is represented by $\:{a}_{m\text{*}}$. The LAC values of the mono energy CT image for material m, $\:{\mu\:}_{E1,m\text{*}}$, and $\:{\mu\:}_{E2,m\text{*}}$ are defined for basis material. By using given $\:{\mu\:}_{E\text{*},m\text{*}}$, and $\:{\mu\:}_{E\text{*}}$, $\:{a}_{m\text{*}}$ can be solved using the least square method to generate the material map. To generate more than three material maps, we generated a triangle set composed of $\:{\mu\:}_{E\text{*},m\text{*}}$, as shown in Fig. 2. First, find the triangle (among 0, 1, and 2) to which the LAC of a mono-energy pair belongs, and then create a material map using the LAC of the material that comprises the triangle. If the LAC does not belong to any triangle, we select the closest triangle to the LAC of the energy pair based on the distance to the center point of the triangle and calculate the material map. Through this process, it is possible to generate multi-material decomposition maps. The triangle set for MD Plus can be changed to correspond to the user’s clinical application. For example, if the material maps of contrast agent, blood, and brain are needed, the triangle can be composed of an LAC pair of these materials.

### Quantitative evaluation

**Fig. 3:**
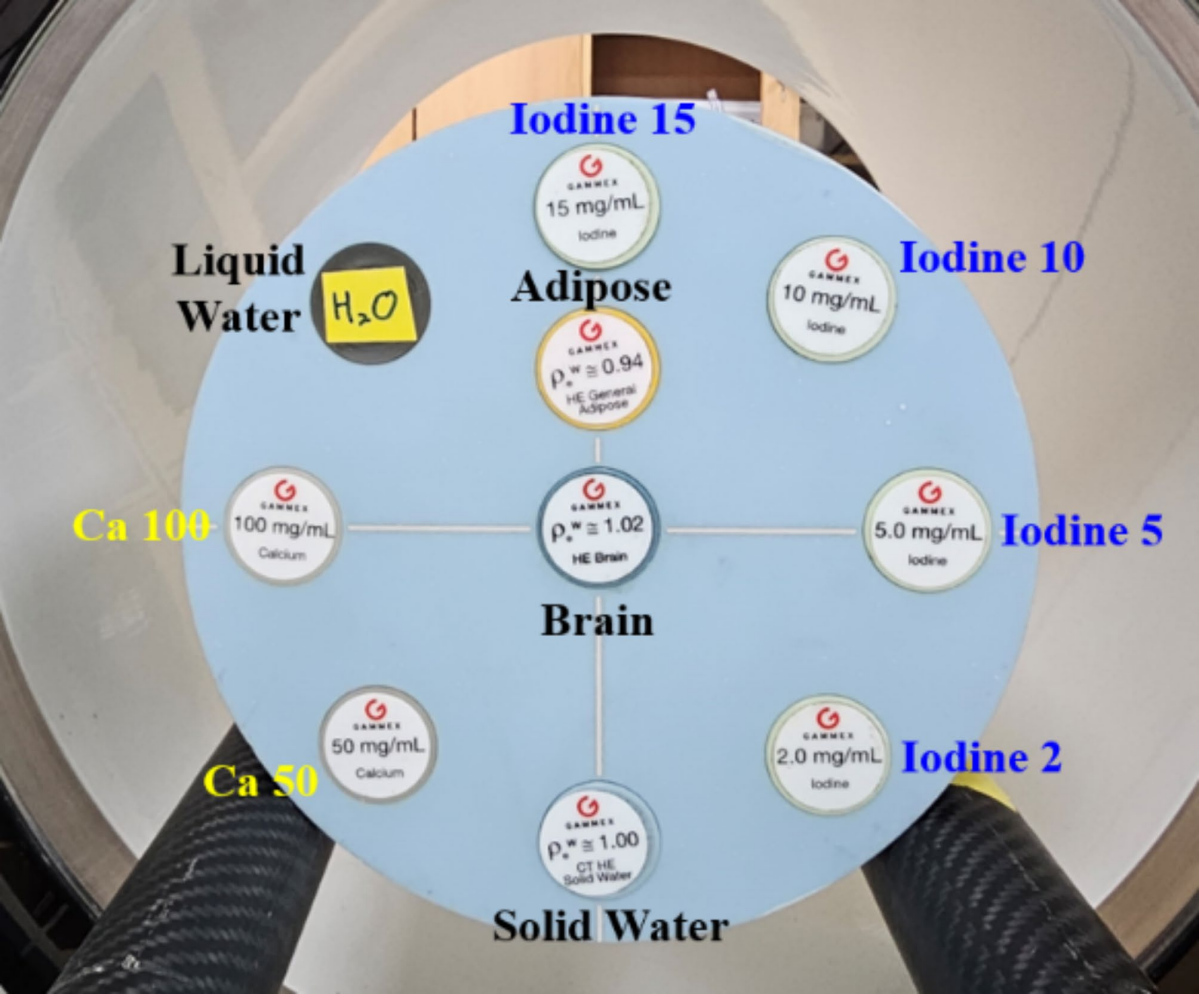
The Gammex multi-energy phantom’s composition for validation of MD Plus algorithm.

For validation of our MD Plus algorithm with the mobile PCD-CT, we experimented using a Gammex Multi-Energy CT phantom (model 1492, Sun Nuclear Corporation, USA), containing different concentrations of solid iodine, different concentrations of calcium, solid water, and human tissue-equivalent inserts. As shown in Fig. 3, the concentrations of solid iodine inserts were 2, 5, 10, and 15 mg/cc, and those of calcium inserts were 50 and 100 mg/cc, respectively. For human tissue equivalent material, high equivalent brain inserts were used. We compared the results of the Gammex phantom with the MD Plus algorithm and that of the basic material decomposition algorithm with the least square method (LS). In addition, we investigated the accuracy of the MD Plus algorithm with various conditions using Gammex phantom. First, we evaluated the material decomposition features at different exposure settings: 5, 10, 15, and 20 mA for 2 s (10, 20, 30, and 40 mAs). Second, we compared the material decomposition maps with different locations of contrast agent. For this experiment, we changed the location of calcium 100 mg/cc or iodine 10 mg/cc insert from edge to center on the phantom.

### Clinical application

To prove the material characterization of neuro-patients in ICU, our MD Plus algorithm was applied to the neuro-images of patients. The CT images of patients were acquired at Massachusetts General Hospital on the mobile PCD-CT system. The patients were scanned at 120 kVp and 40 mAs (37.16mGy). First, we applied our MD Plus algorithm to the neuro-images of traumatic brain injury patients at the ICU without the administration of a contrast agent. The ability to differentiate between iodine and hemorrhage was evaluated. The material maps were decomposed as blood, brain with iodine, and air map. Second, we also applied our MD Plus algorithm to the neuro-images of patients at the ICU who had acute ischemic stroke treated with mechanical thrombectomy. The iodine contrast was used during the angiography, and due to the blood-brain barrier in the stroke area, the potential damage may result in prolonged contrast staining, similar to intracranial hemorrhage in CT number (HU). We investigated the differentiation ability of staining of iodine contrast agent from secondary brain hemorrhage.

### VNC and bone removal

In addition, we extended our algorithm to two clinical applications: VNC and bone removal. The VNC refers to a process in which the enhancing effect of contrast agent has been removed from CT images^12,15^. Our VNC method replaces the volume of contrast agent in each voxel with the same volume of brain tissue. To reconstruct the linear attenuation coefficient (LAC) of the VNC image, the LAC of three-energy channel images of PCD-CT and MD Plus maps is required. In terms of MD Plus maps, there are two maps: one is for the iodine material detection ($\:{x}_{d}$, using non-negative least square), and the other ($\:{x}_{r}$, using least square solver) is for the reconstruction of VNC image. We create an LAC matrix of channel data corresponding to the map ($\:{M}_{c}$) and an LAC matrix that replaces iodine with the brain ($\:{M}_{nc}$).

3 $$\:{M}_{c}=\:\left[\begin{array}{ccc}{\mu\:}_{Ioidne}\left({E}_{ch0}\right)&\:{\mu\:}_{Blood}\left({E}_{ch0}\right)&\:{\mu\:}_{Calcium}\left({E}_{ch0}\right)\\\:{\mu\:}_{Ioidne}\left({E}_{ch1}\right)&\:{\mu\:}_{Blood}\left({E}_{ch1}\right)&\:{\mu\:}_{Calcium}\left({E}_{ch1}\right)\\\:{\mu\:}_{Ioidne}\left({E}_{ch2}\right)&\:{\mu\:}_{Blood}\left({E}_{ch2}\right)&\:{\mu\:}_{Calcium}\left({E}_{ch2}\right)\end{array}\right]$$

4 $$\:{M}_{nc}=\:\left[\begin{array}{ccc}{\mu\:}_{Brain}\left({E}_{ch0}\right)&\:{\mu\:}_{Blood}\left({E}_{ch0}\right)&\:{\mu\:}_{Calcium}\left({E}_{ch0}\right)\\\:{\mu\:}_{Brain}\left({E}_{ch1}\right)&\:{\mu\:}_{Blood}\left({E}_{ch1}\right)&\:{\mu\:}_{Calcium}\left({E}_{ch1}\right)\\\:{\mu\:}_{Brain}\left({E}_{ch2}\right)&\:{\mu\:}_{Blood}\left({E}_{ch2}\right)&\:{\mu\:}_{Calcium}\left({E}_{ch2}\right)\end{array}\right]$$

When reconstructing the VNC images, if $\:{x}_{d}\left({w}_{Iodine}\right)$ is above the threshold, $\:{M}_{nc}$ is used. Otherwise, $\:{M}_{c}$ is used.

5 $$\left[ {\begin{array}{*{20}l} {VNC_{{ch0}} } \\ {\:VNC_{{ch1}} } \\ {\:VNC_{{ch2}} } \\ \end{array} } \right] = \left\{ {\begin{array}{*{20}c} {x_{r} \: \cdot \:\:M_{{nc}} \:,} & {if\:\:x_{d} \left( {w_{{Iodine}} } \right) > threshold} \\ {x_{r} \: \cdot \:\:M_{c} ,} & {otherwise} \\ \end{array} } \right.,$$

where $$\:\:{x}_{d}=\:\left[\begin{array}{c}\:{w}_{Iodine}\\\:\:{w}_{Blood}\\\:\:{w}_{Calcium}\end{array}\right].$$

**Fig. 4:**
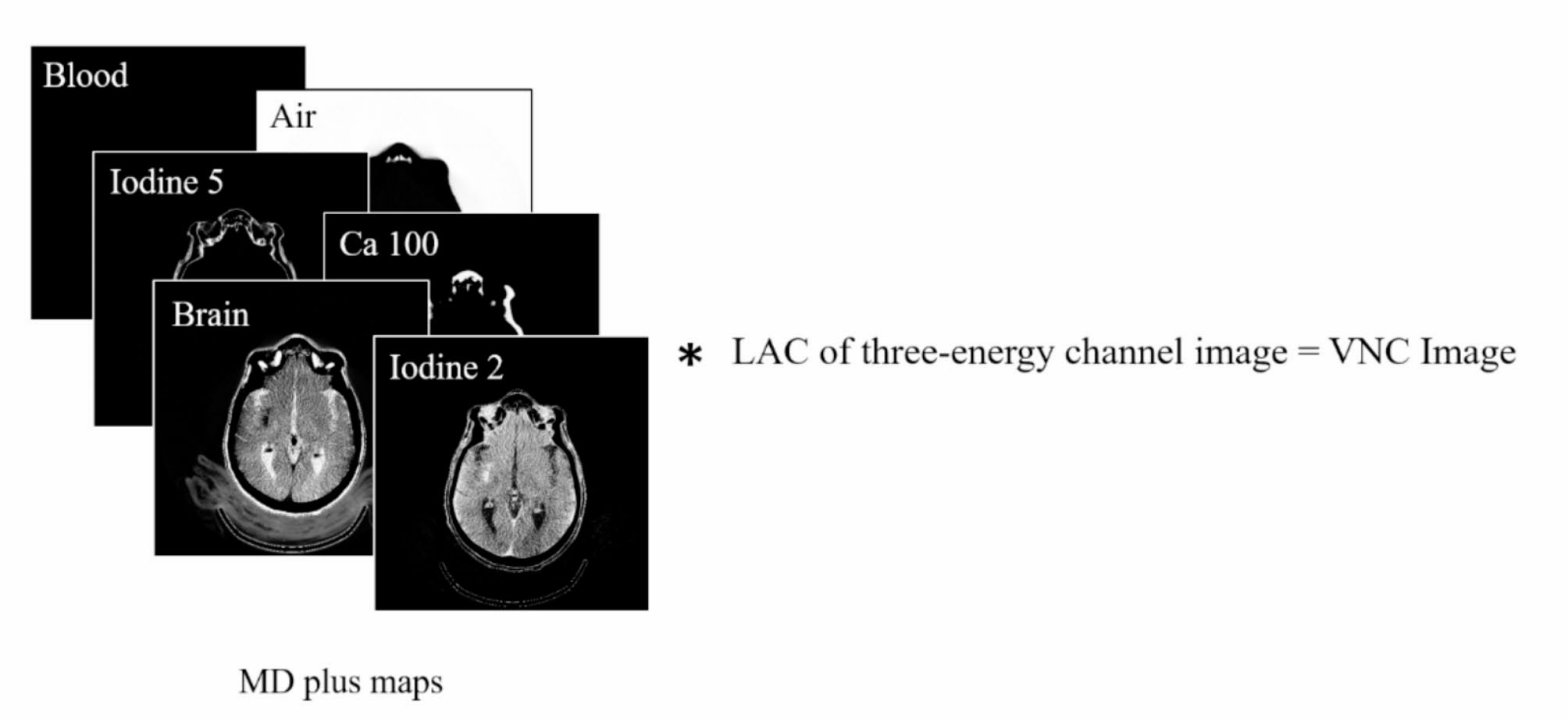
The overview of VNC reconstruction.

**Fig. 5:**
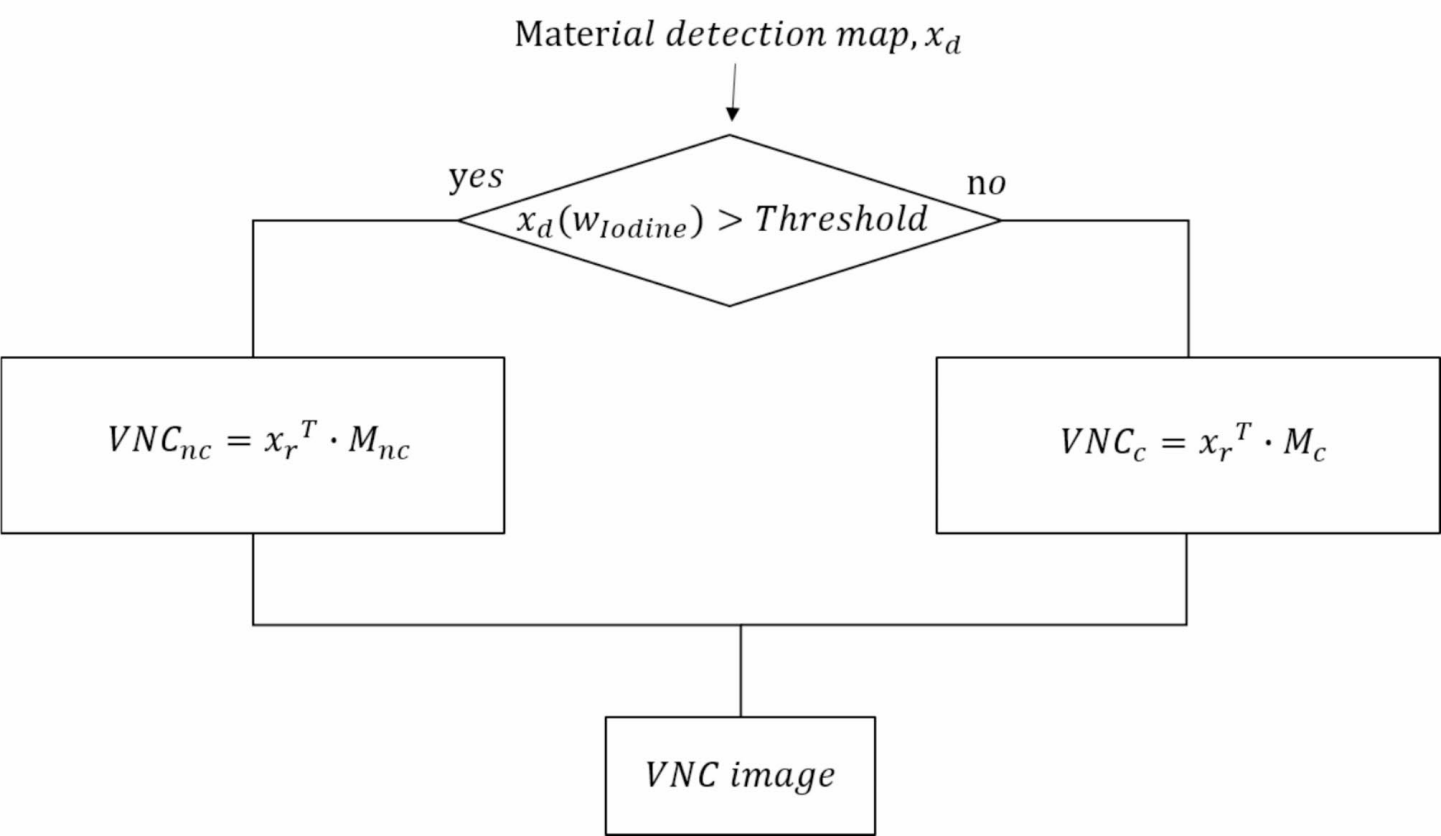
The flow diagram illustrates the process of VNC.

Figure 4 illustrates the overview of the VNC reconstruction, and Fig. 5 shows the flow diagram illustrating the process of VNC.

**Fig. 6:**
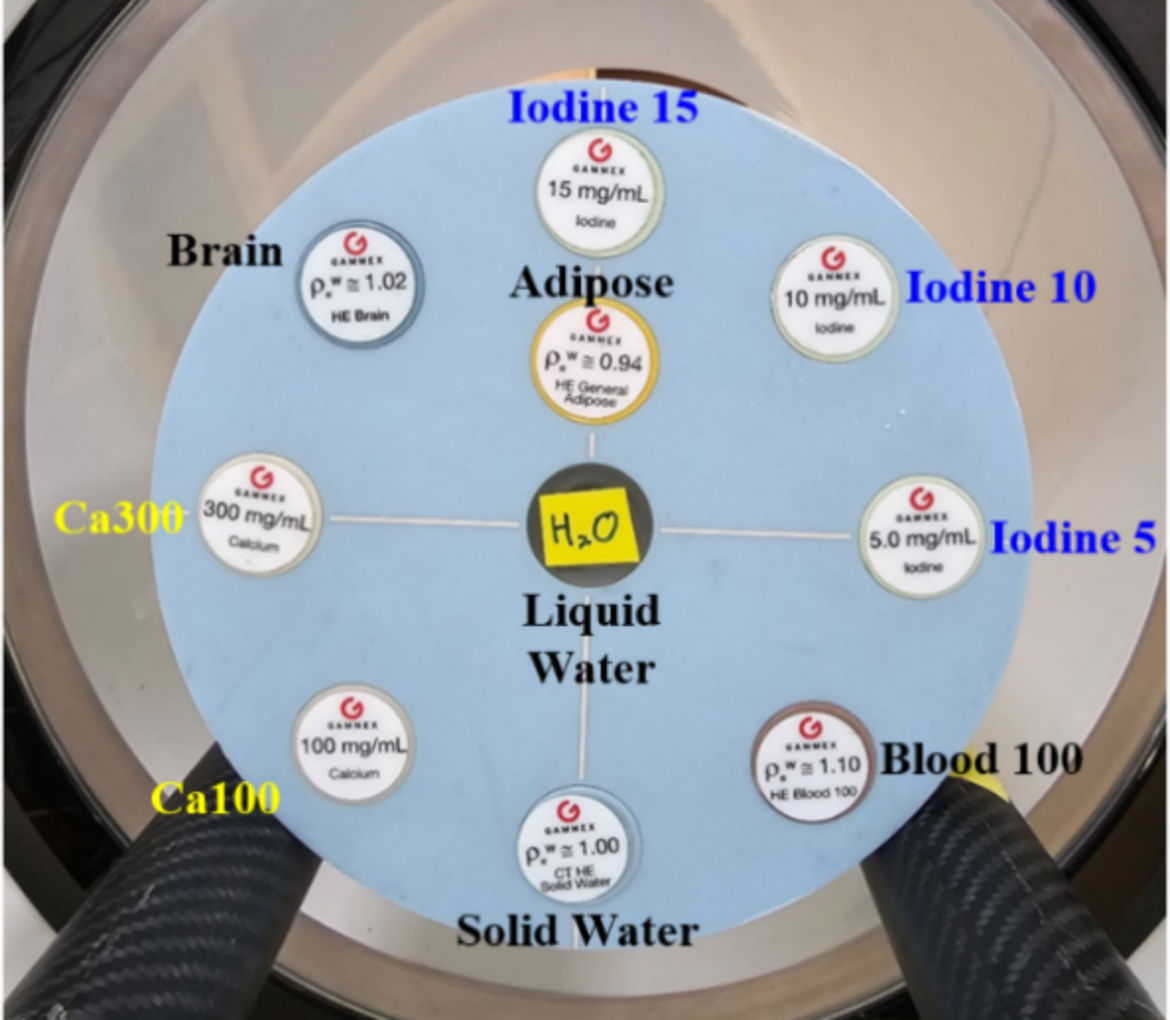
The Gammex multi-energy phantom’s composition for VNC.

**Fig. 7:**
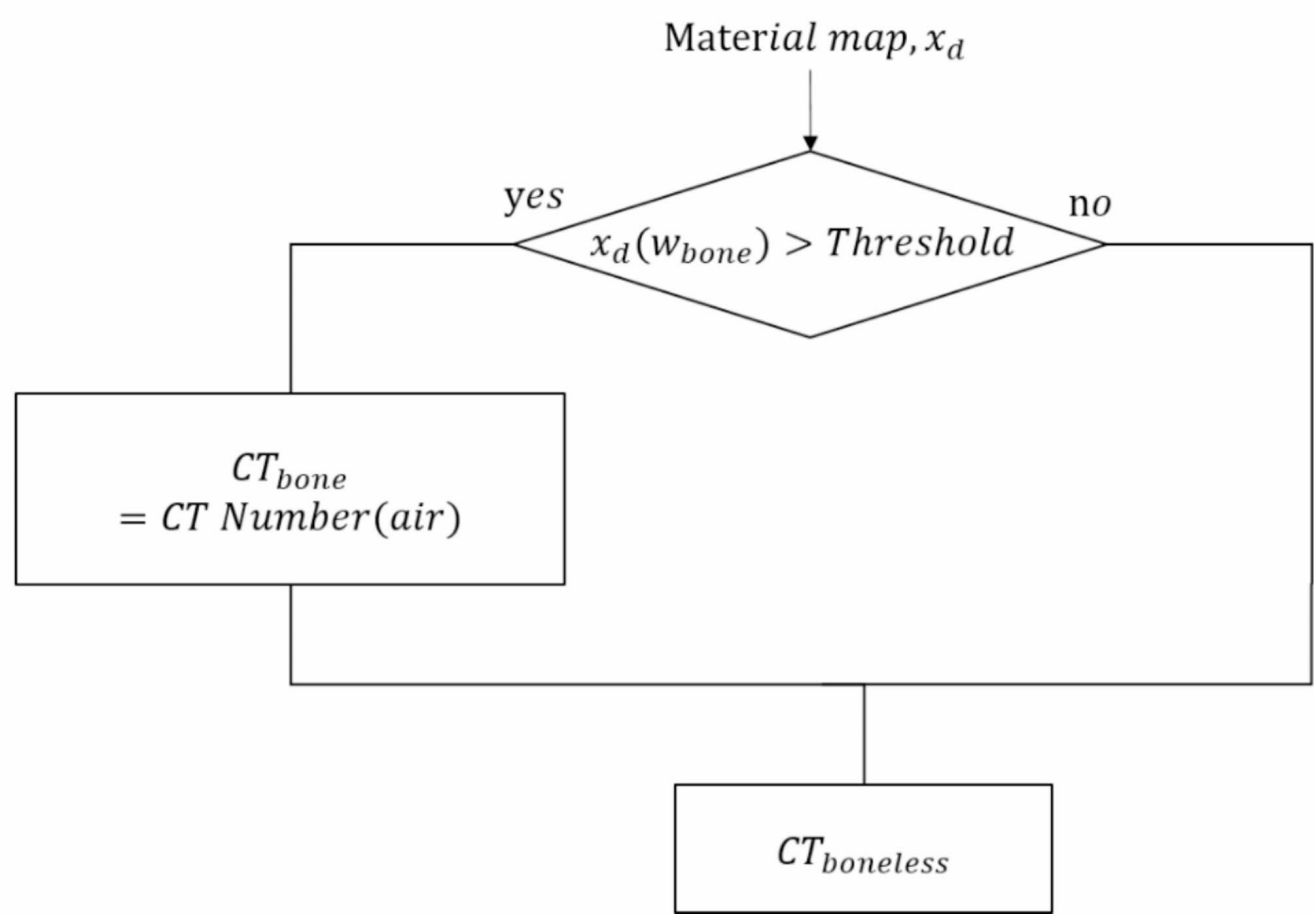
The flow diagram illustrates the process of bone removal.

**Fig. 8:**
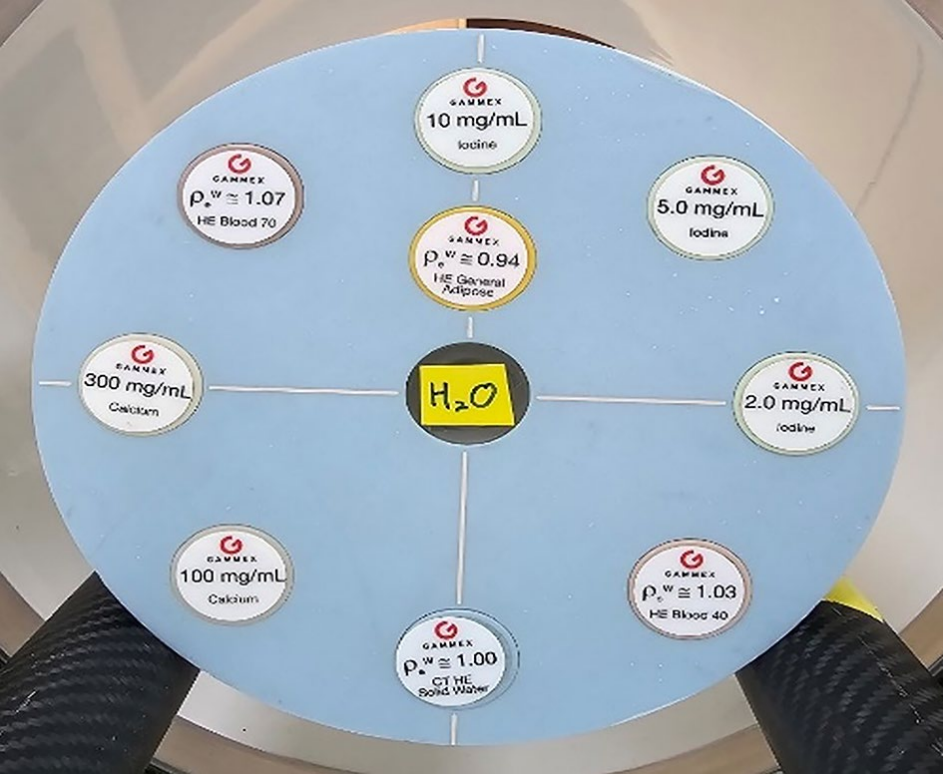
The Gammex multi-energy phantom’s composition for bone removal.

In order to validate our VNC method, we performed experiments with the Gammex phantom. Also, the optimal threshold value for VNC was determined as 0.6 through phantom studies. The composition of inserts is shown in Fig. 6. We also applied our VNC method to the neuro-images of ICU patients treated with mechanical thrombectomy. The second extended clinical application of the MD Plus algorithm is bone removal. The bone removal images can be generated by using the MD Plus maps. Our bone removal method replaces the volume of bone in each voxel with the same volume of air. Figure 7 shows the flow diagram illustrating the process of bone removal. The Gammex phantom was used to validate bone removal method and to set the optimal threshold as 0.3. The composition of inserts in the Gammex phantom is illustrated in Fig. 8. Our bone removal algorithm was also applied to the human brain CT image with PCD-CT.

Informed consent was waived from the Mass General Brigham (MGB) Institutional Review Board (IRB) due to the retrospective design. All experiments were performed following the relevant regulation and the guidelines. All protocols used in the experiment chosen and approved by the Massachusetts General Hospital.

## Results

### Quantitative evaluation

**Fig. 9:**
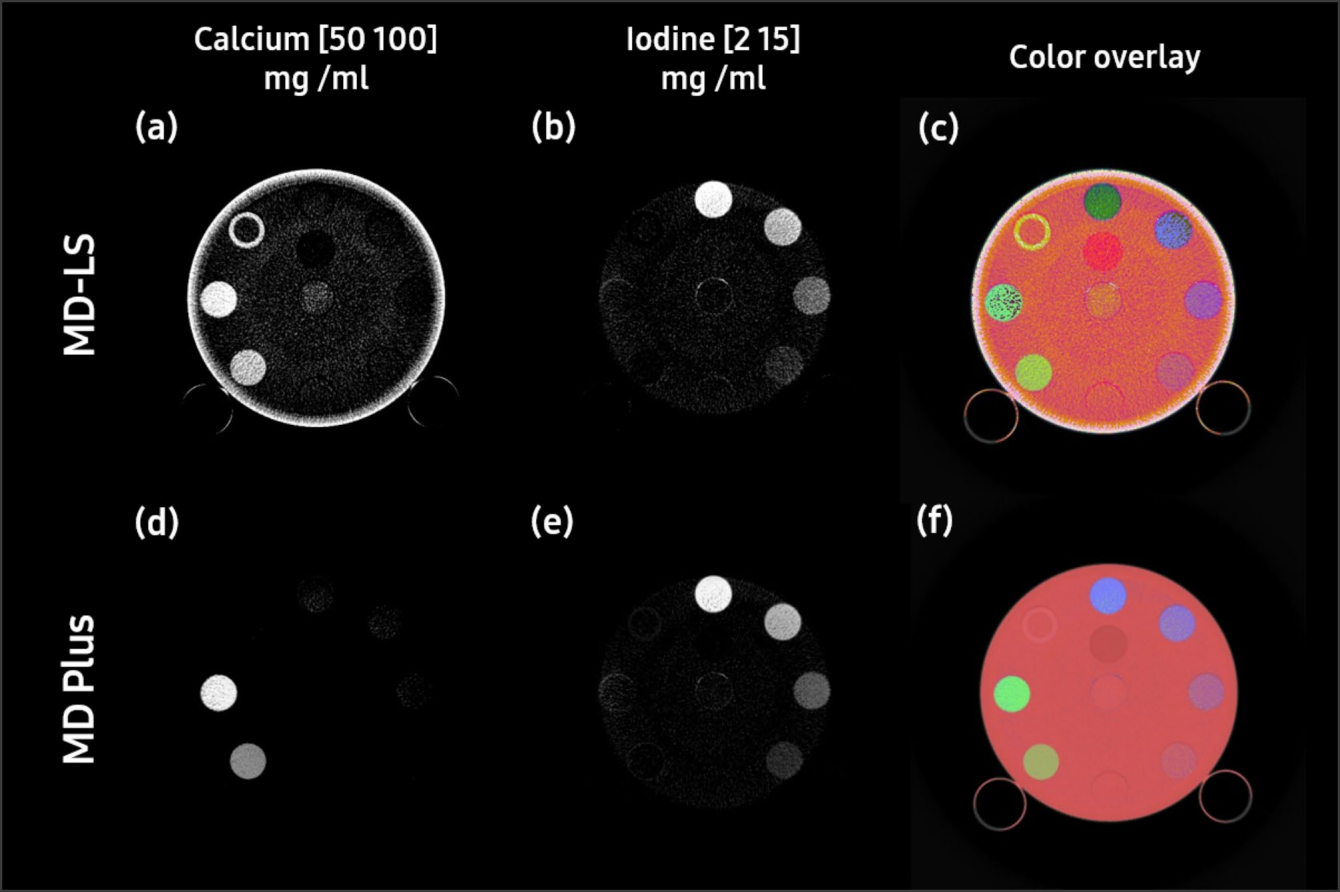
The comparison of material decomposition maps with least square (MD-LS) and MD Plus. The results of MD-LS are (**a**–**c**), and that of MD Plus are (**d**–**f**). The calcium map with MD-LS and that with MD Plus are (**a**) and (**d**), respectively. The iodine map with MD-LS and that with MD Plus are (**b**) and (**e**), respectively. The overlay of the material decomposition maps for MD-LS and for MD-plus are (**c**) and (**f**), respectively.

Our MD Plus algorithm’s performance in material decomposition was compared with basic material decomposition using the LS method (MD-LS). As shown in Fig. 9, the calcium and iodine were more clearly decomposed with our MD Plus algorithm compared with MD-LS. Moreover, the artifacts of the edge on the calcium map using MD-LS disappeared when using our MD Plus algorithm. In addition, as illustrated in Table 1, the CNR of MD plus was higher than that of MD-LS for all of insert material.

**Table 1:** The comparison of contrast-to-noise ratio (CNR) with least square (MD-LS) and MD Plus.

Map	Insert material	CNR	
		MD-LS	MD Plus
Calcium 100 mg/mL	Calcium 100 mg/mL	5.607	13.988
	Calcium 50 mg/mL	4.367	14.364
Iodine 15 mg/mL	Iodine 15 mg/mL	7.007	15.808
	Iodine 10 mg/mL	5.319	9.220
	Iodine 5 mg/mL	2.932	5.037
	Iodine 2 mg/mL	1.120	2.000

**Fig. 10:**
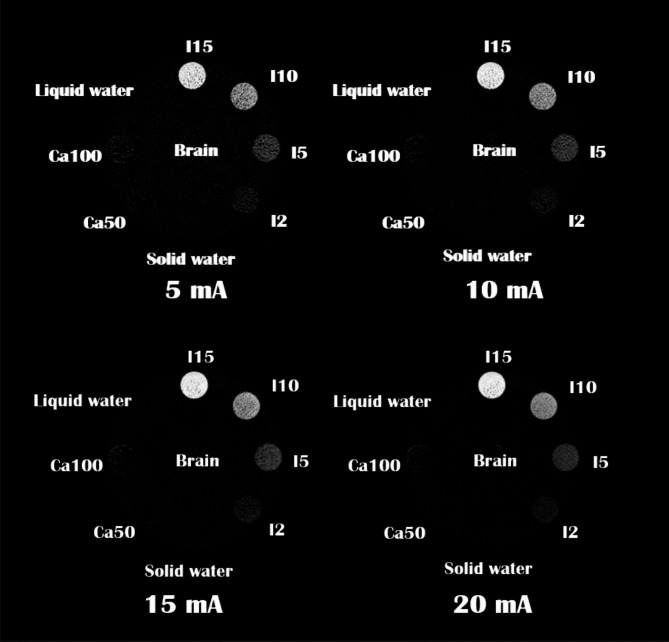
The iodine maps for different exposure settings are 5, 10, 15, and 20 mA for 2 s (10, 20, 30, and 40 mAs). The display window/level is 15/7.5 mg/cc.

The accuracy of our MD Plus algorithm was quantitatively investigated using various conditions using a Gammex phantom. Figure 10 displays the material decomposition maps for iodine at different exposures. The grey levels of all four images were very similar.

**Fig. 11:**
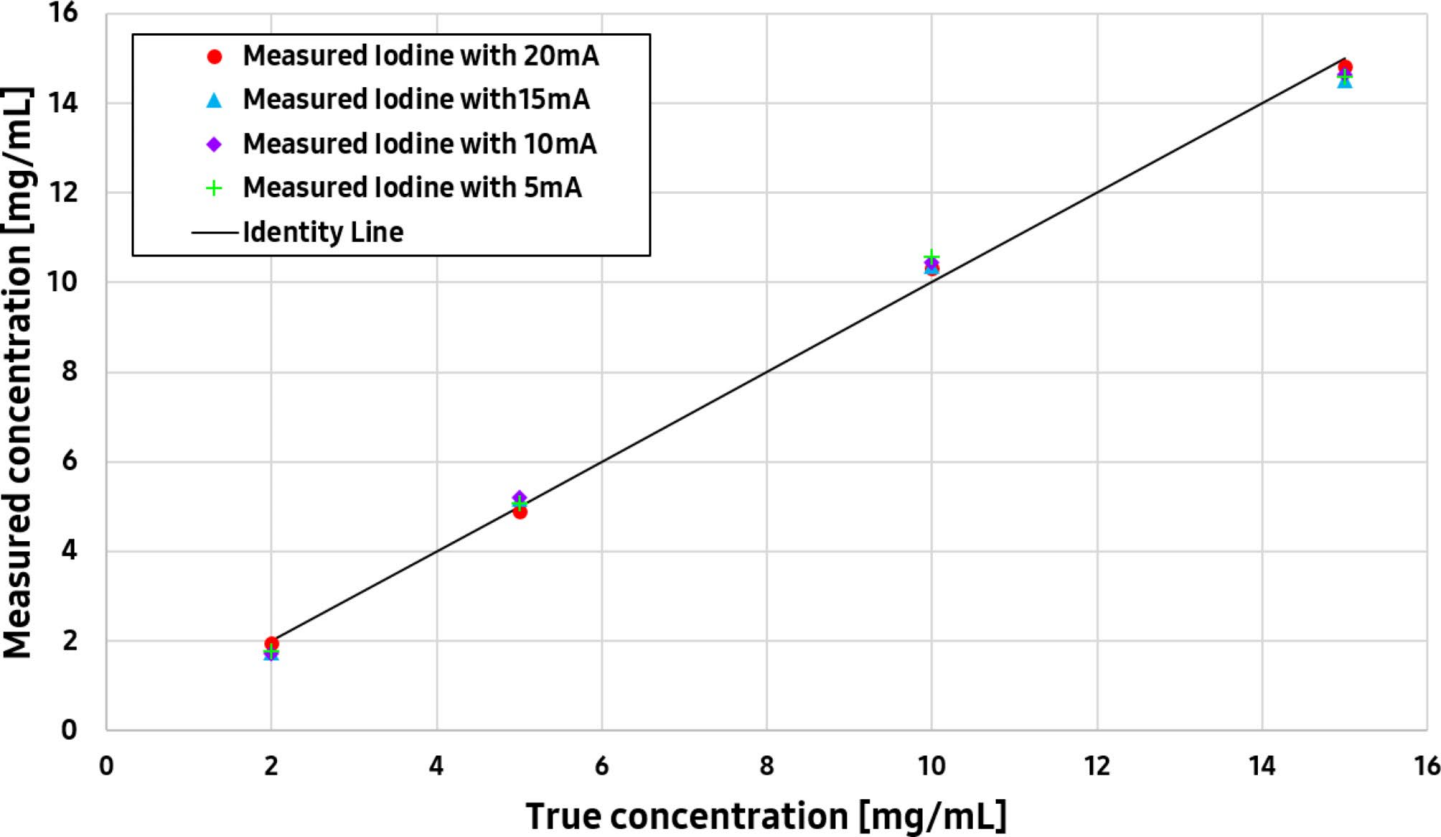
Comparison of the measured iodine concentration with actual concentration for different exposure settings: 5, 10, 15, 20 mA.

The measured iodine concentrations of each exposure setting were compared to the ground truth, namely the absolute known concentration in each insert, and errors were calculated. A linear fit was performed, and the coefficient of determination (R^2^) was reported. Figure 11 shows a good match with the ground truth for all exposure settings. A linear relationship was observed between the measured and actual iodine concentrations for the PCD CT systems. The coefficient of determinations (R^2^) for 5, 10, 15, and 20 mA were 0.994, 0.995, 0.996, and 0.998 respectively.

**Fig. 12:**
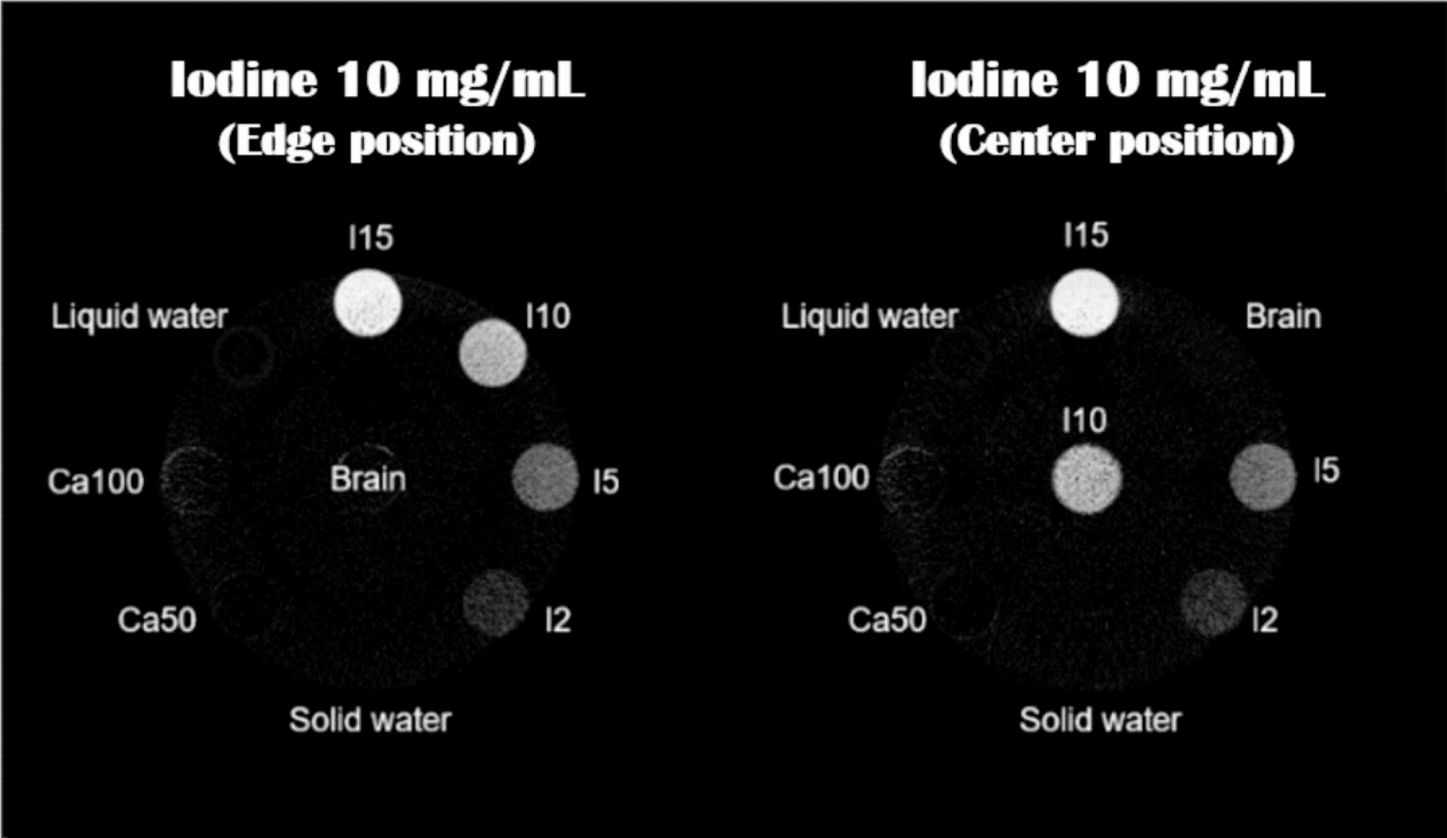
The iodine maps for different locations of Iodine 10 mg/cc. Display window/level: 15/7.5 mg/cc.

Figure 12 shows the material decomposition map of iodine with different iodine insert locations. The material maps were comparable, and the difference ratio was 3.644%.

**Fig. 13:**
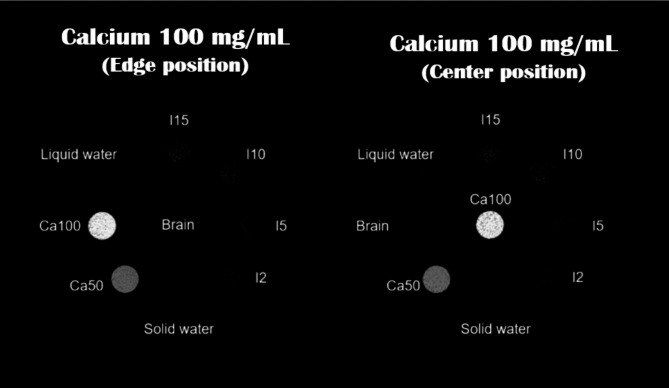
The calcium maps for different locations of calcium 100 mg/cc. Display window/level: 100/50 mg/cc.

Figure 13 shows the material decomposition map of calcium with different iodine insert locations. As shown in Fig. 13, the material maps were comparable. The quantitative values of calcium 100 mg/cc from each material map were also evaluated, and the difference ratio was 3.534%.

### Clinical evaluation of MMD

**Fig. 14:**
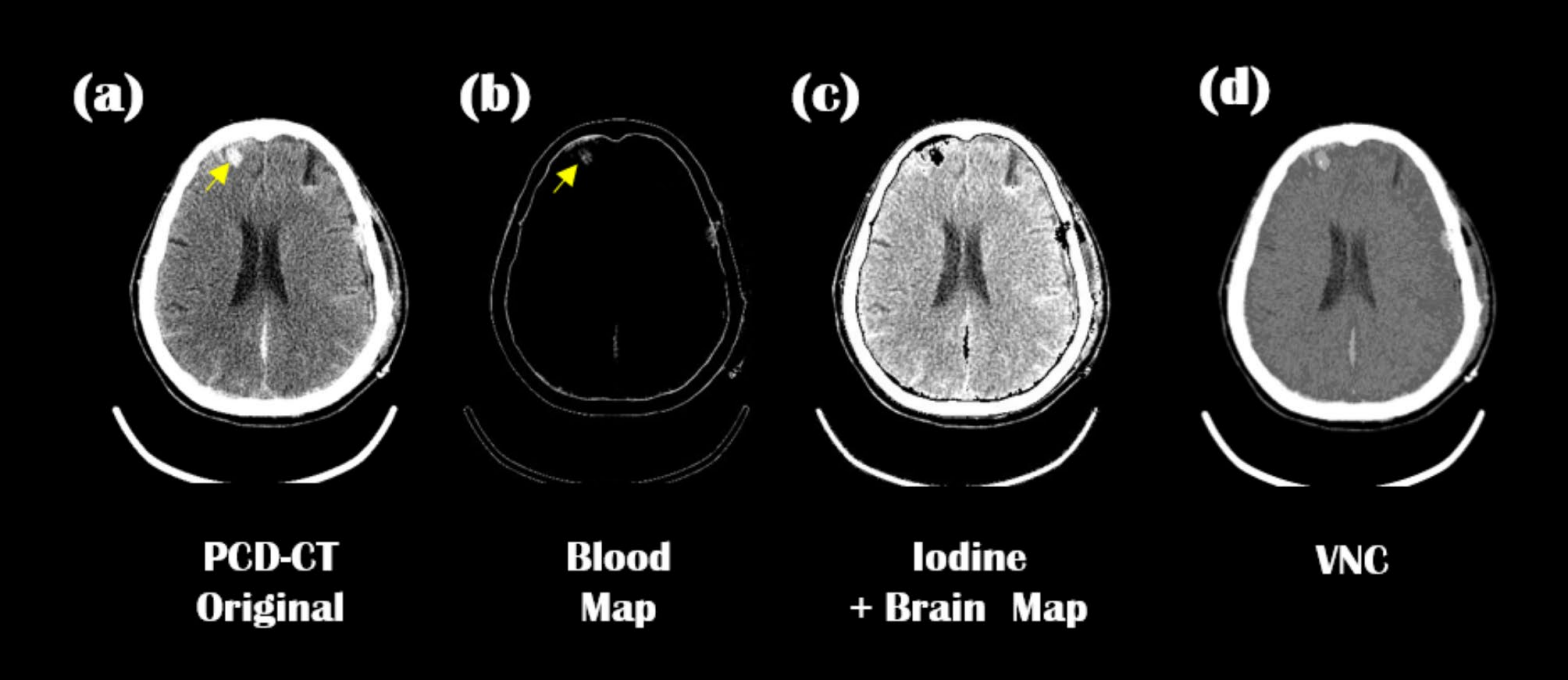
The hyperdense area on the PCD-CT image, and corresponding to material decomposition maps; PCD-CT original image (**a**), blood map (**b**); iodine combined with brain map (**c**); VNC (**d**).

**Fig. 15:**
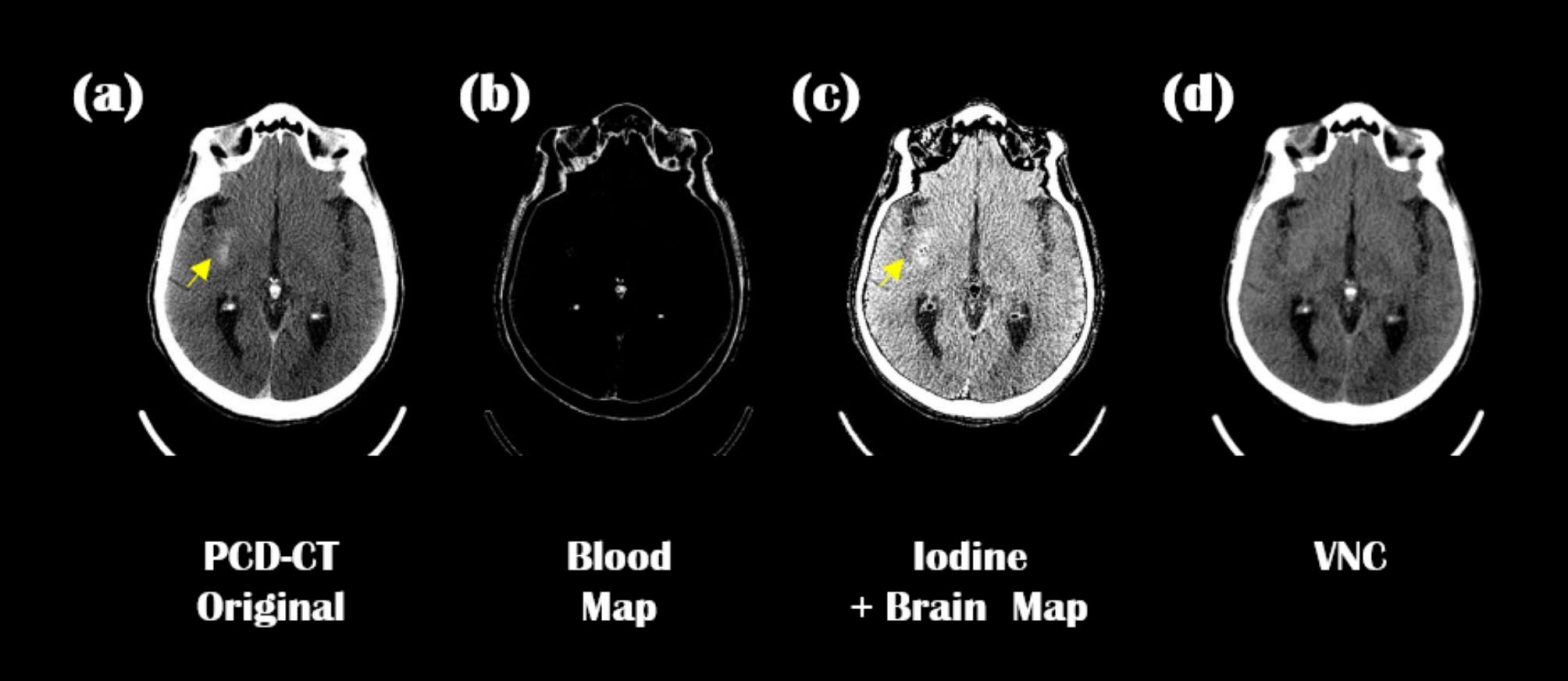
The hyperdense area on the PCD-CT image, and corresponding to material decomposition maps; PCD-CT original image (**a**), blood map (**b**); iodine combined with brain map (**c**); VNC (**d**).

Our MD Plus algorithm was applied to the neuro-images of traumatic brain injury patients at the ICU. Figures 14 and 15 show two representative neuro-images of patients presenting hyperdense areas (yellow arrows) in PCD-CT original images; this may result in prolonged staining of the contrast agent or secondary hemorrhage. In Fig. 14, the hyperdense area (yellow arrow) was visible in the material decomposition map of blood. In contrast, the same area was seen as hypodense in the iodine contrast agent map combined with the brain map. Thus, according to the MD Plus algorithm results, we concluded that the hyperdense area was a hemorrhage, not an iodine contrast agent. In Fig. 15, the hyperdense area (yellow arrow) was seen in the iodine contrast agent map combined with the brain map, while the same area was not highlighted in the blood map. This result indicated that the hyperdense area was an iodine contrast agent. With the MD Plus algorithm using the mobile PCD-CT, it was possible to distinguish iodine staining from brain hemorrhage on material decomposition maps.

### VNC and bone removal

**Fig. 16:**
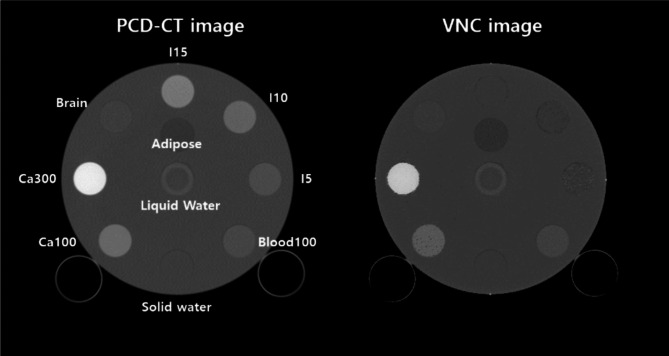
The PCD-CT image and its VNC image. The threshold for VNC was set as 0.6.

**Fig. 17:**
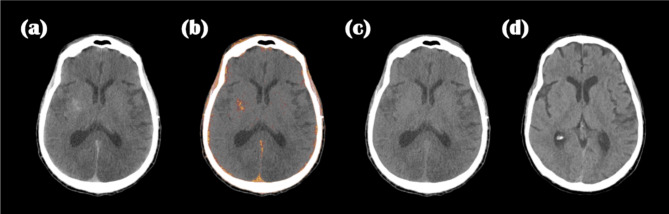
The neuro-images of patients with acute ischemic stroke treated using mechanical thrombectomy. (**a**) The original images of mobile PCD-CT, (**b**) iodine overlay map, (**c**) VNC image, and (**d**) follow-up non-contrast CT (NCCT) image. The threshold of VNC was set as 0.6.

For the extended clinical application, the VNC of the Gammex phantom was generated, as shown in Fig. 16, and the regions of iodine contrast were replaced with brain tissue. Figure 17 (c) shows the results of the VNC and the disappearance of the iodine contrast agent. In addition, the VNC image is similar to the follow-up non-contrast CT (NCCT) image (Fig. 17 (d)).

**Fig. 18:**
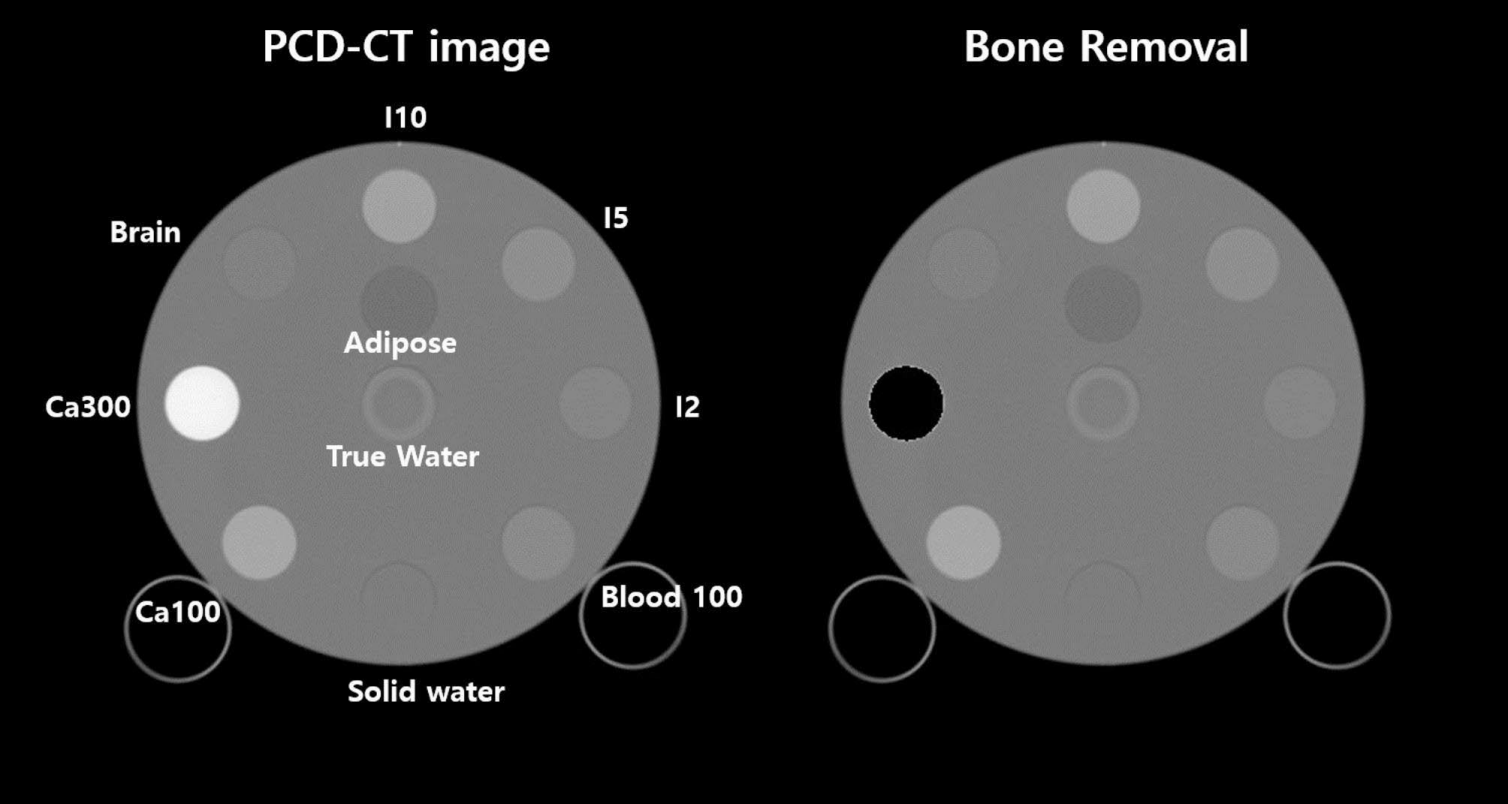
The Gammex phantom image applied the bone removal method. The threshold for bone removal was set as 0.3. The high density of the insert (calcium 300 mg/cc) disappeared.

**Fig. 19:**
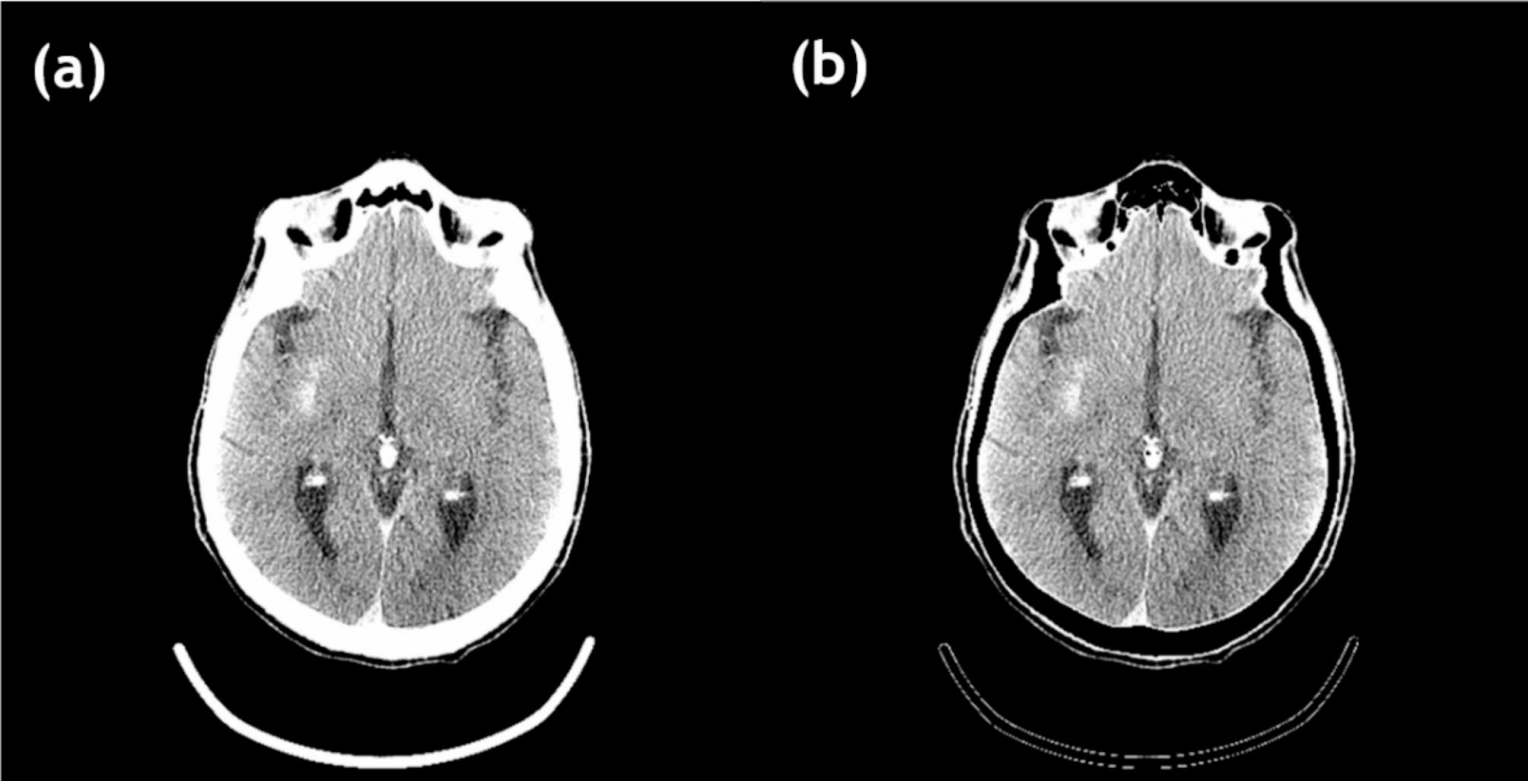
The human brain image from PCD-CT (**a**) and the images applied bone removal method (**b**). The threshold was set as 0.3. The region of bone in the human brain was removed.

For the validation of our bone removal algorithm, the bone removal image of the PCD-CT with Gammex phantom was generated, as shown in Fig. 18. As we assumed that the insert of calcium 300 mg/cc is bone, the insert disappeared. Figure 19 shows the human brain CT image with PCD-CT and its bone removal image. The results illustrate that the regions of bone disappeared.

## Discussion

Our results demonstrate that the MD Plus algorithm with the mobile PCD-CT can be a valuable tool for early differentiation of hemorrhage and iodine contrast agent. Conventional CT often cannot distinguish hemorrhage from iodine extravasation following reperfusion therapy for acute ischemic stroke^13^ since the materials with similar attenuation can be difficult to distinguish on conventional CT. However, with the mobile PCD-CT using the MD Plus algorithm, it should be possible to distinguish iodine staining from brain hemorrhage on material decomposition maps, which is essential in the clinical scenario concerning early and accurate decision-making. This is also critical for the applicability of antithrombotic treatment in ischemic stroke^1^. Figure 17 (a) shows that the hyperdense areas could be mistaken for the secondary hemorrhage in the original PCD image. However, the iodine overlay maps in conjunction with VNC images showed that the staining of the iodine contrast agent was visible in the hyperdense regions, which can be used to identify intracranial iodine definitively and thus potentially aid in early detection^2^. Moreover, as illustrated in Figs. 15 and 16, the capability of providing the material decomposition maps, including blood map, can help to identify subarachnoid hemorrhage (SHA) adjacent bone or spot signs^16^.

The results from the quantitative analysis showed that the MD Plus algorithm with the mobile PCD-CT has spectral capabilities, providing reliable multi-energy images for material decomposition regardless of the exposure settings or conditions. The coefficient of determinations (R^2^) was above 0.994 for all exposure settings, having a good match with the ground truth. Moreover, the difference ratios for different iodine or calcium insert locations were below 3.644%.

In addition, our results of the VNC image were similar to the follow-up NCCT image, as shown in Fig. 17. The VNC derived from the MD Plus algorithm with the mobile PCD-CT can be an alternative to NCCT imaging and may provide additional information, such as accurate detection of intracranial hemorrhage (ICH) after mechanical thrombectomy in patients with acute ischemic stroke^1^. VNC intends to provide users with images equivalent to NCCT images acquired without a contrast agent^12,15^. This provides a potential radiation dose reduction for patients and minimizes the risk of radiation-induced malignancy^17^. Additional benefits of VNC include simplified clinical protocols and improved workflow from both the patients’ and clinicians’ perspectives^18^.

Moreover, as Figs. 18 and 19 demonstrate, MD can provide the bone removal image plus algorithm with the mobile PCD-CT. Bone removal in CT could improve the detection of small intracranial hemorrhages, particularly those adjacent to bone, by removing bone that can interfere with the visualization of small acute hemorrhages. The bone removal application is also helpful in detecting acute intracranial hemorrhagic lesions such as subdural, epidural, or contusional hematomas and acute venous thrombosis. In an emergency such as head trauma, bone removal CT can help detect small acute hemorrhagic lesions^10^. However, as our current bone removal algorithm considers bones with high densities, it has limitations when removing bones with varying densities. Thus, further studies are required to enhance the performance of the bone removal algorithm.

Regarding the mobility of the portable PCD-CT, accessible transportation is helpful for imaging critically ill neuro-patients with high risks when transferred to a fixed CT suite. As the mobile PCD-CT can be brought closer to the bedside, the risks associated with transport can be reduced. Additionally, the scanner can be quickly and efficiently moved around the hospital, so the overall time needed for imaging can be reduced^4^.

We acknowledge the following limitations. First, the number of patients included in this work was relatively low, and additional studies with a higher number of cases are required to confirm our results. Secondly, follow-up imaging was performed using a different imaging technique at predefined time intervals. Therefore, we plan to conduct a study based on a larger group of patients, including follow-up imaging at a predefined time and using the same imaging modality. Third, the quantitative analysis of VNC and bone removal is needed to validate algorithms. For example, the VNC has to be verified for different iodine concentrations because the concentration of iodine solution can vary with different clinical applications. Also, bone removal for different densities of calcium or bones has to be validated since the range of bone density is wide in the human body. Lastly, comparing material decomposition performance with different spectral modalities, including dual-energy CT, is needed. Thus, we plan to compare the material differentiation between dual-energy CT and the mobile PCD-CT. In order to take advantage of the PCD characteristics, that is, energy-bin separation, MD Plus with three separate energy bins has to be performed. Therefore, we are investigating the MD Plus with three energy bins. Using the current version of the MD Plus algorithm, the LACs of mono-energy for materials have to be known or provided in the NIST database. On the other hand, when the MD Plus with three energy bins is possible, no prior information is needed, such as LACs of mono-energy for materials.

To our knowledge, no previous study investigated using mobile PCD-CT to differentiate hemorrhage from iodine contrast in neuroradiological applications. Our results provide essential reference information for task-based neuroimaging studies using PCD-CT and can be utilized in establishing new protocols for mobile PCD-CT. Moreover, these results offer insights into the potential of multi-energy imaging with PCD-CT.

## Conclusion

Our quantitative analysis of multi-energy phantom and clinical results of neuro-patients at ICUs demonstrate that the MD Plus algorithm with mobile PCD-CT allows for clear visualization and quantification of hemorrhage or iodine contrast in the brain. Moreover, our VNC and bone removal proved helpful and may aid in the early detection of hemorrhagic lesions. Furthermore, the mobile PCD-CT, which can be brought to the patient at any location, may lead to decreased transportation-related morbidity and improved rapid decision-making in the ICU.

## Data Availability

The datasets generated during or analyzed during the current study are available from the corresponding author upon reasonable request.
